# Supplementary figures and images for: Parallels and contrasts between the cnidarian and bilaterian maternal-to-zygotic transition are revealed in Hydractinia embryos
Source: PLoS Genet. 2023 Jul 13;19(7):e1010845. doi: 10.1371/journal.pgen.1010845 (PMC10368294; doi:10.1371/journal.pgen.1010845)

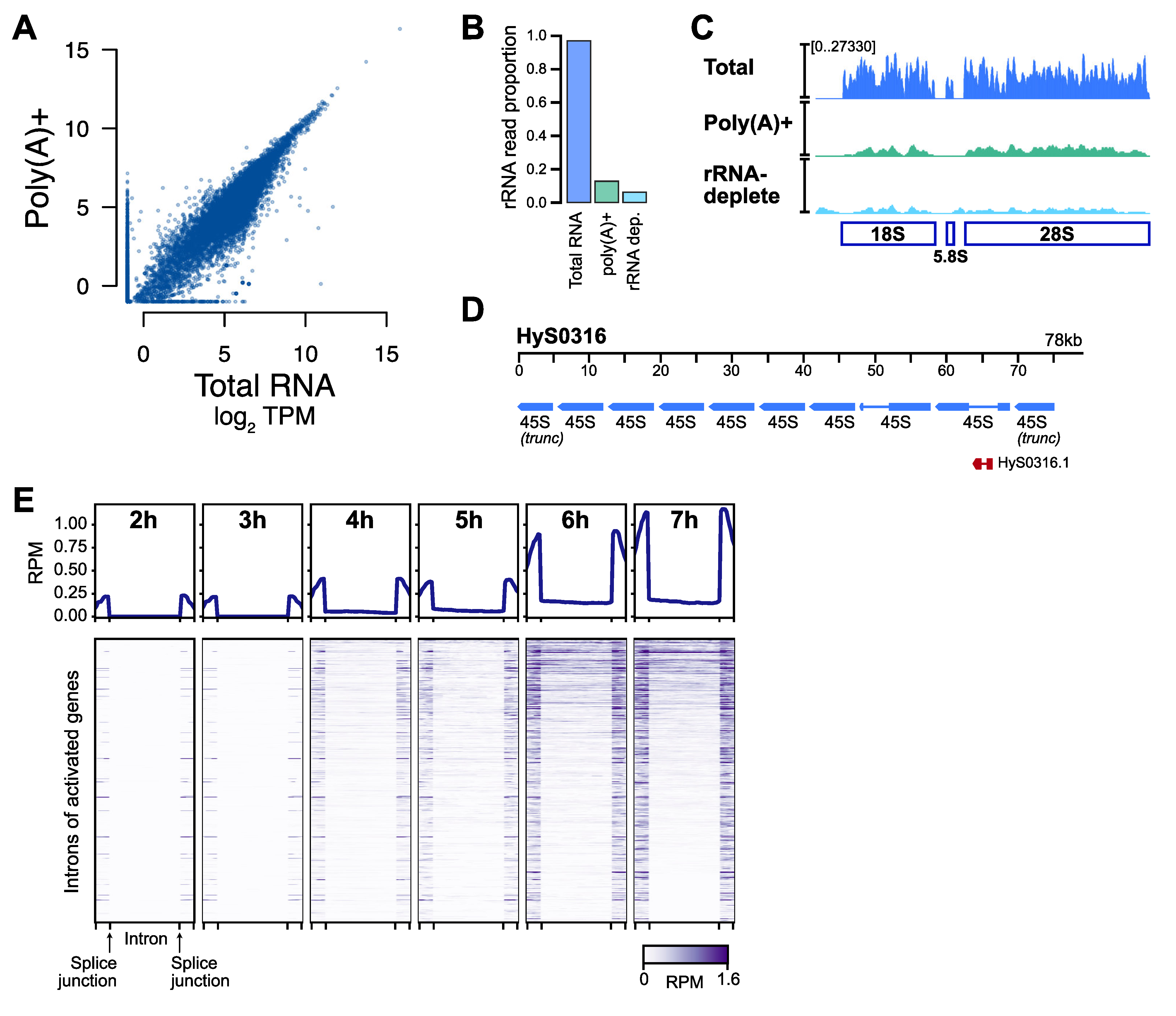

Supplement: S1 Fig — (A) Biplot comparing total RNA-seq (no selection) to poly(A)+ selected RNA-seq. (B) Proportion of sequencing reads mapping to rRNA without selection, with poly(A)+ selection, and with rRNA depletion at 1 hour post fertilization (h.p.f.). (C) Browser tracks over the composite 45S rRNA locus showing RNA-seq coverage in the different selection strategies. (D) Browser track showing a predicted array of 45S genes on the genome scaffold HyS0316. (E) Heatmaps showing intronic RNA-seq coverage of activated genes over time. RPM = reads per million. (TIF) [file pgen.1010845.s001.tif]

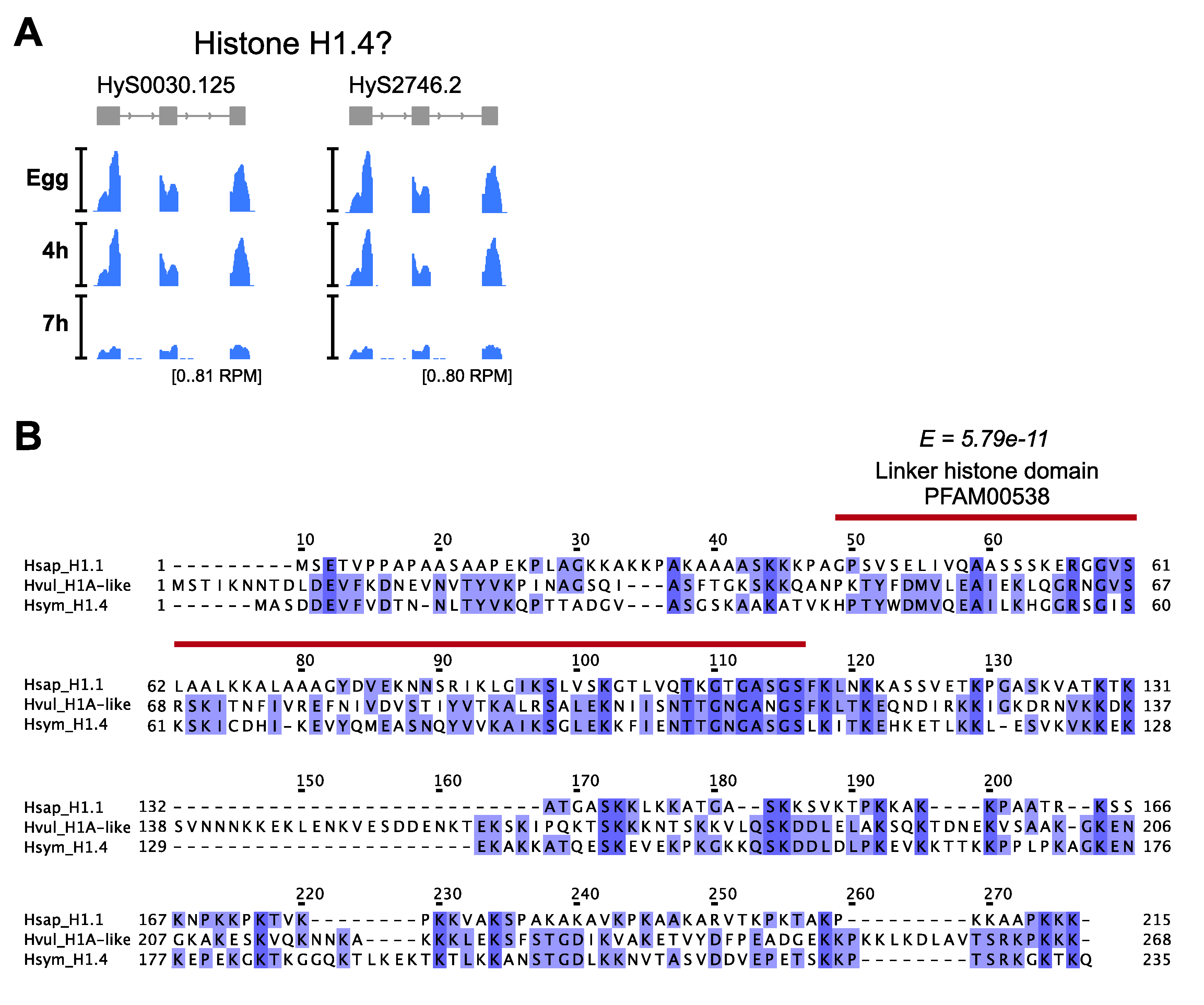

Supplement: S2 Fig — (A) Browser tracks showing two identical novel H1 genes (H1.4) and RNA-seq coverage over time. (B) Multiple alignment of the amino acid sequences of human H1.1 (top), Hydra vulgaris H1A-like (middle) and the novel H. symbiolongicarpus H1.4 (bottom). The CD-Search annotated linker histone domain is marked in red. (TIF) [file pgen.1010845.s002.tif]

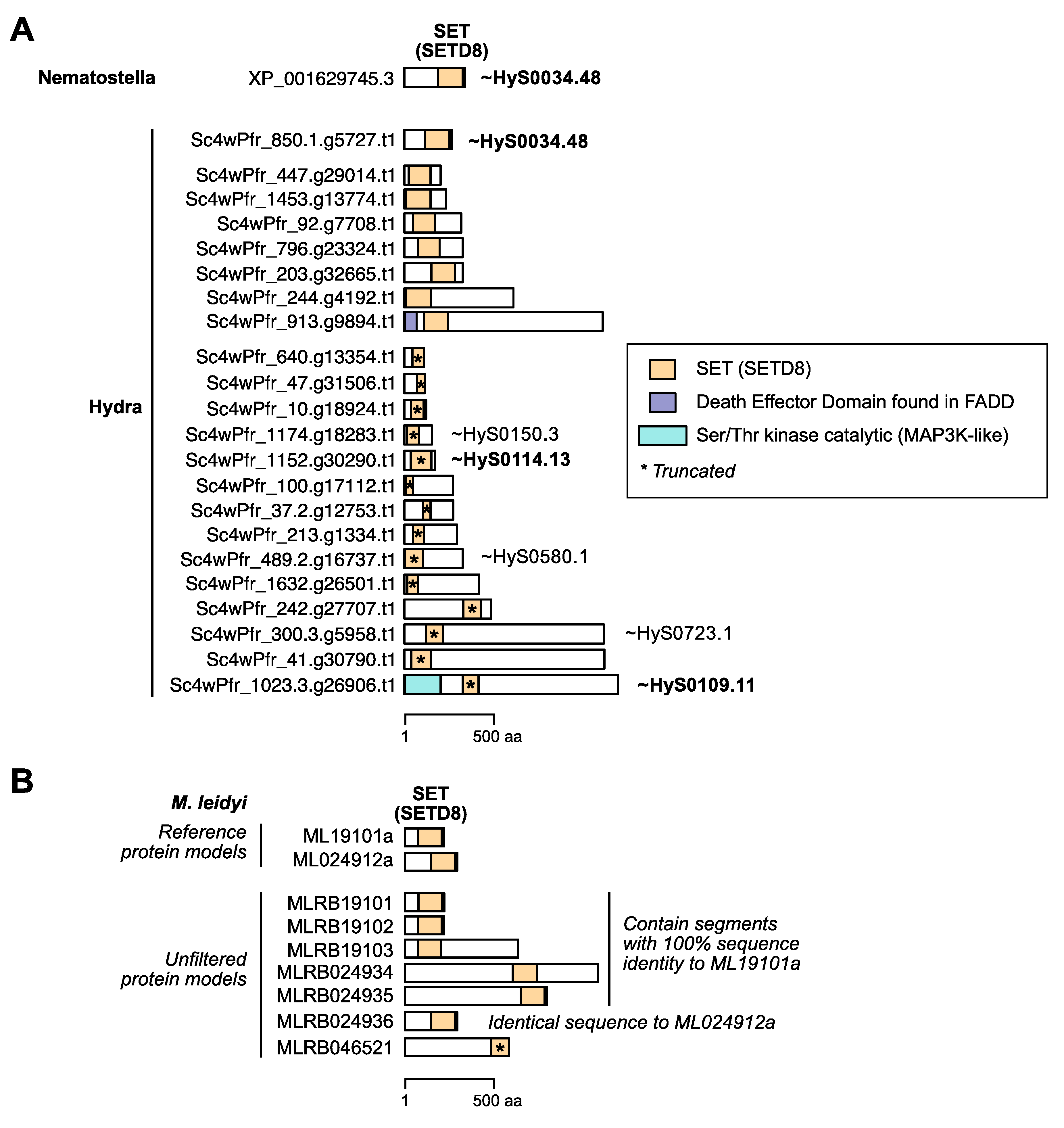

Supplement: S3 Fig — (A) Protein domain schematics for predicted H4K20 methyltransferases in other cnidarians, showing positions of the SETD8 SET domain (orange), as predicted by CD-Search. Reciprocal best BLAST hits to Hydractinia H4K20 methyltransferases are annotated to the right; embryonic-expressed genes are bolded. (B) Predicted H4K20 methyltransferases in M. leidyi. (TIF) [file pgen.1010845.s003.tif]

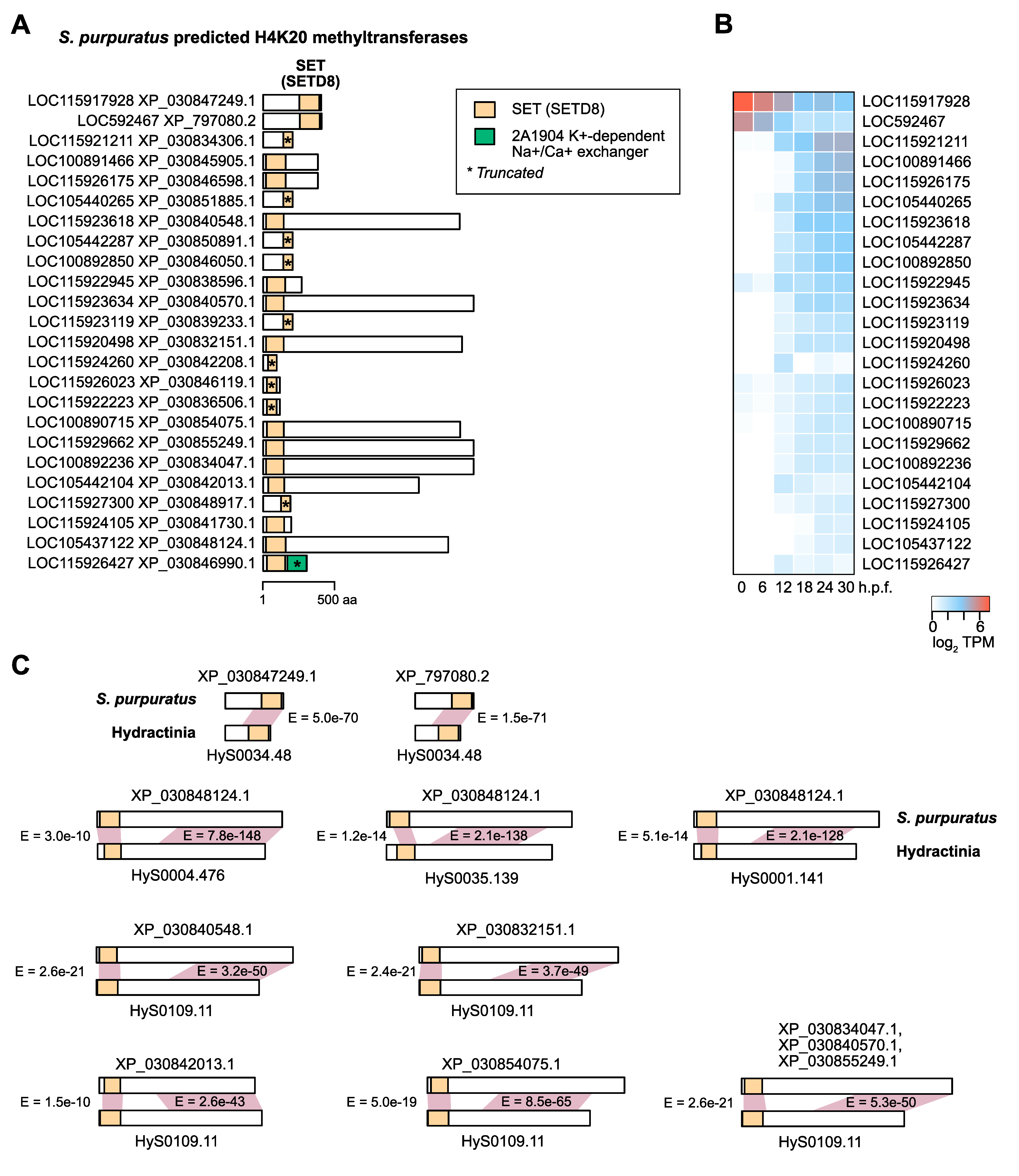

Supplement: S4 Fig — (A) Protein domain schematics for predicted H4K20 methyltransferases in sea urchin, showing positions of the SETD8 SET domain (orange), as predicted by CD-Search. (B) Heatmap showing expression levels of the H4K20 methyltransferases over sea urchin development, using the data of Khor et al 2021. h.p.f. = hours post fertilization. (C) Schematics showing BLAST similarity between sea urchin (top in each pair) and Hydractinia H4K20 methyltransferases. BLAST E-values for each significant high-scoring pair (pink shaded regions) are annotated. SET domains are orange. Three nearly identical sea urchin genes (lower right) have the same degree of BLAST similarity to HyS0109.11, so only one representative schematic is shown. (TIF) [file pgen.1010845.s004.tif]

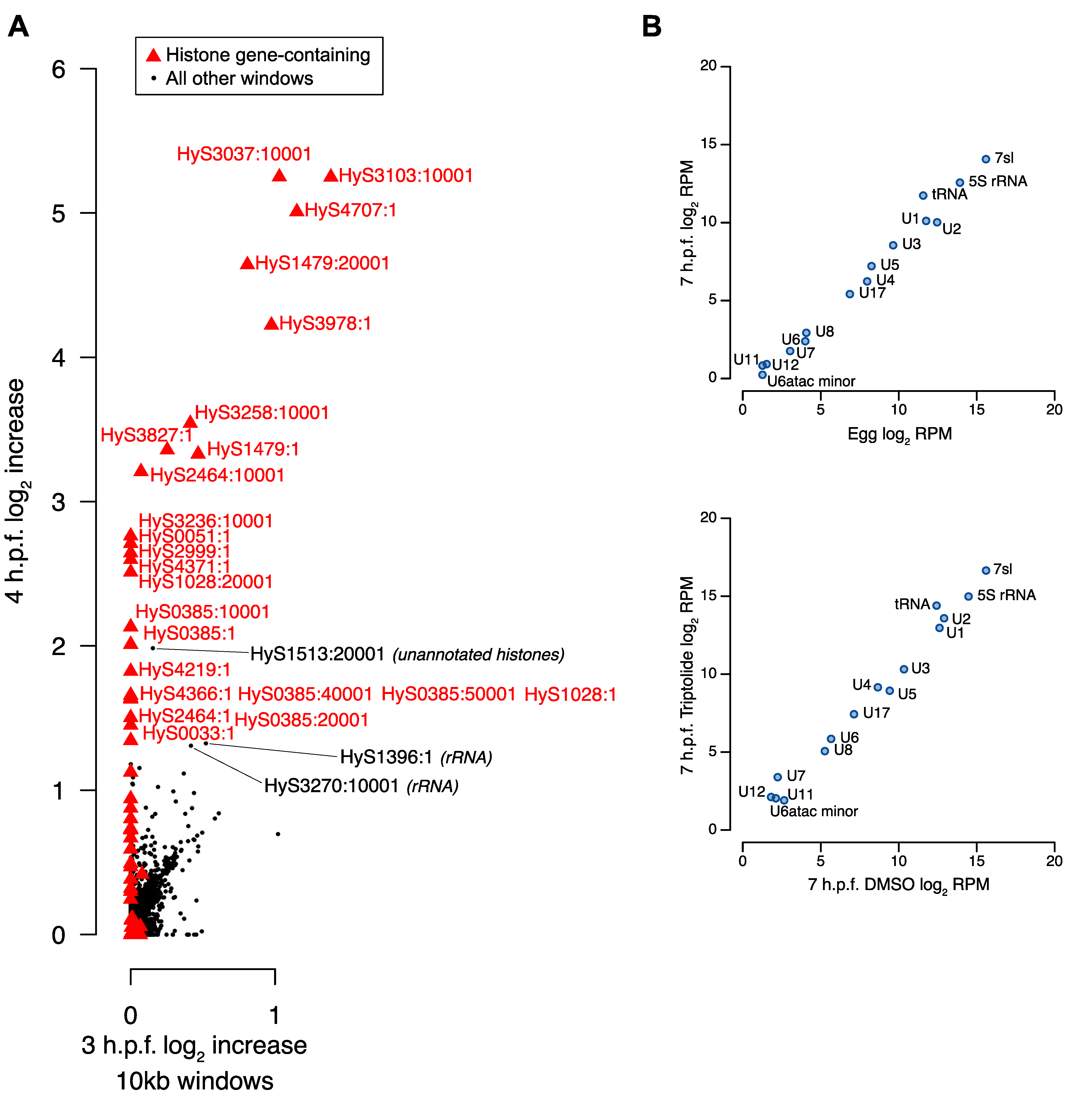

Supplement: S5 Fig — (A) Comparison of activation levels of 10-kb windows tiled across the genome at 3 hours post fertilization (h.p.f.) versus egg (x axis) and 4 h.p.f. versus egg (y axis). Windows with major levels of activation are accounted for by histone genes (red triangles), except for two windows that have predicted ribosomal RNA genes. (B) Biplots of summed expression across the predicted noncoding gene classes uncovered by Stringtie transcriptome assembly. (TIF) [file pgen.1010845.s005.tif]

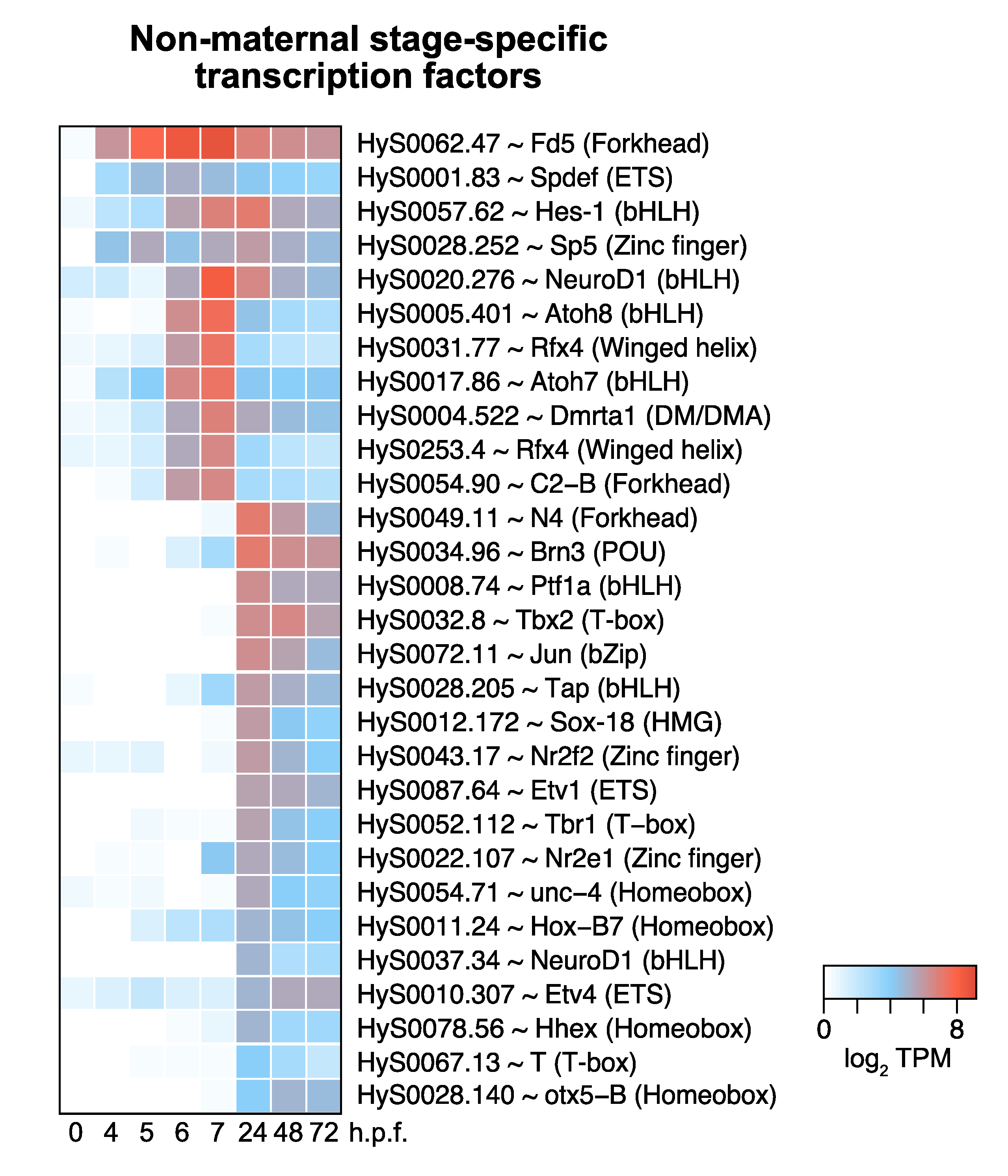

Supplement: S6 Fig — Heatmap showing expression patterns of non-maternal stage-specific transcription factors. Gene names are the best BLAST hit to UniProtKB / SwissProt, DNA binding domains are in parentheses. (TIF) [file pgen.1010845.s006.tif]

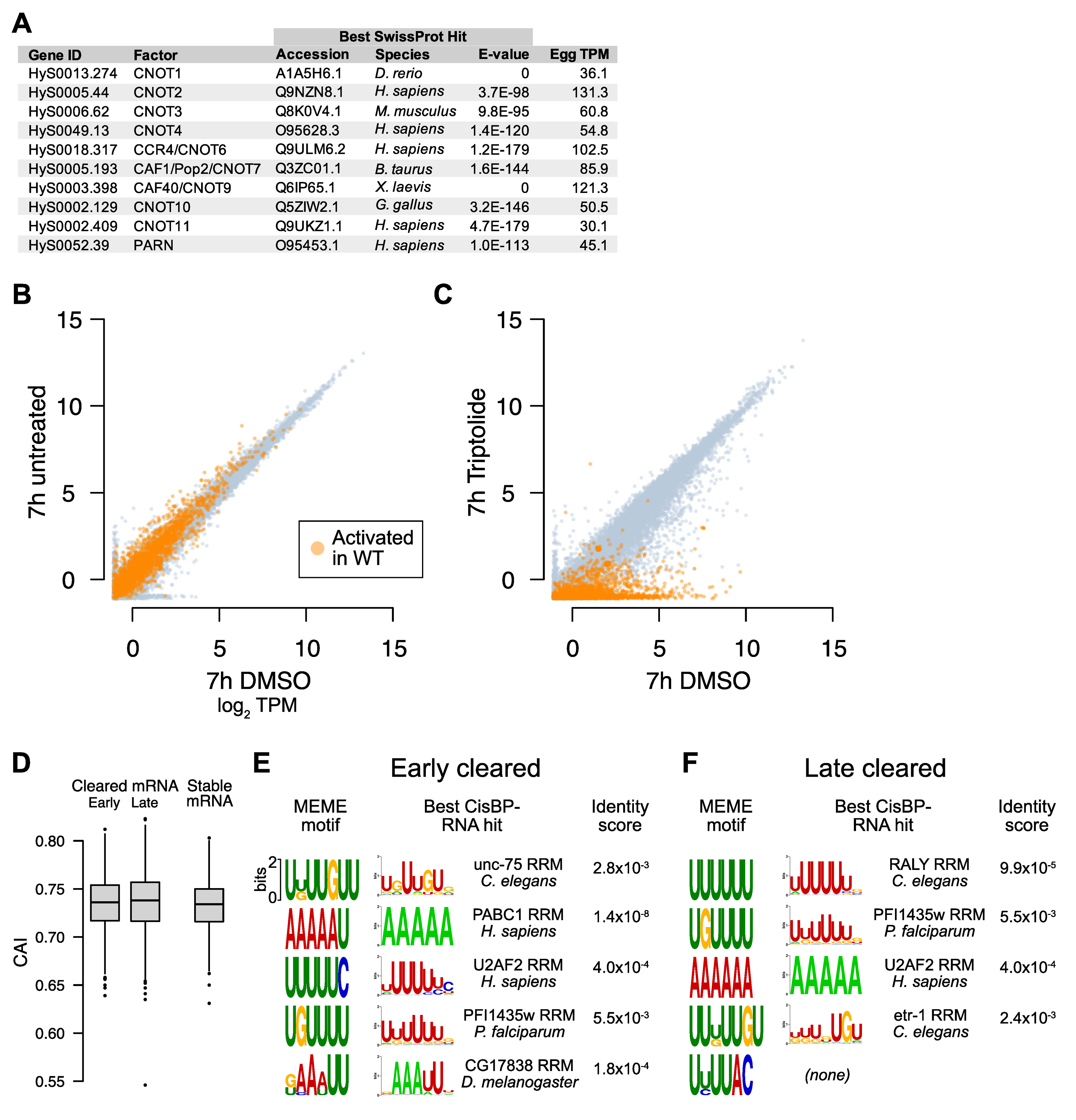

Supplement: S7 Fig — (A) Table of maternal expression levels of predicted deadenylase factors as identified by BLAST search. (B) Biplot showing expression levels in untreated versus DMSO vehicle embryos. Orange points are genes with significant activation over time in wild-type embryos. (C) Biplot showing failed activation of wild-type activated genes (orange) with Triptolide treatment. Two of these genes have prominently higher expression upon Triptolide treatment, HyS0422.6 and HyS4764.1. These are both single-exon genes similar to retrovirus-related reverse transcriptase genes, suggesting that these might actually be transposon sequences and not transcribed by Pol II. Conversely, these transposons may be inhibited by mechanisms requiring genome activation, which would account for their up-expression upon Triptolide treatment. TPM = transcripts per million. (D) Comparison of codon adaptation index (CAI) between cleared mRNA and a set of stable mRNA, showing no significant decreased CAI associated with clearance. (E, F) Top hits from the CISBP-RNA database for each of the MEME motifs enriched in cleared genes. (TIF) [file pgen.1010845.s007.tif]

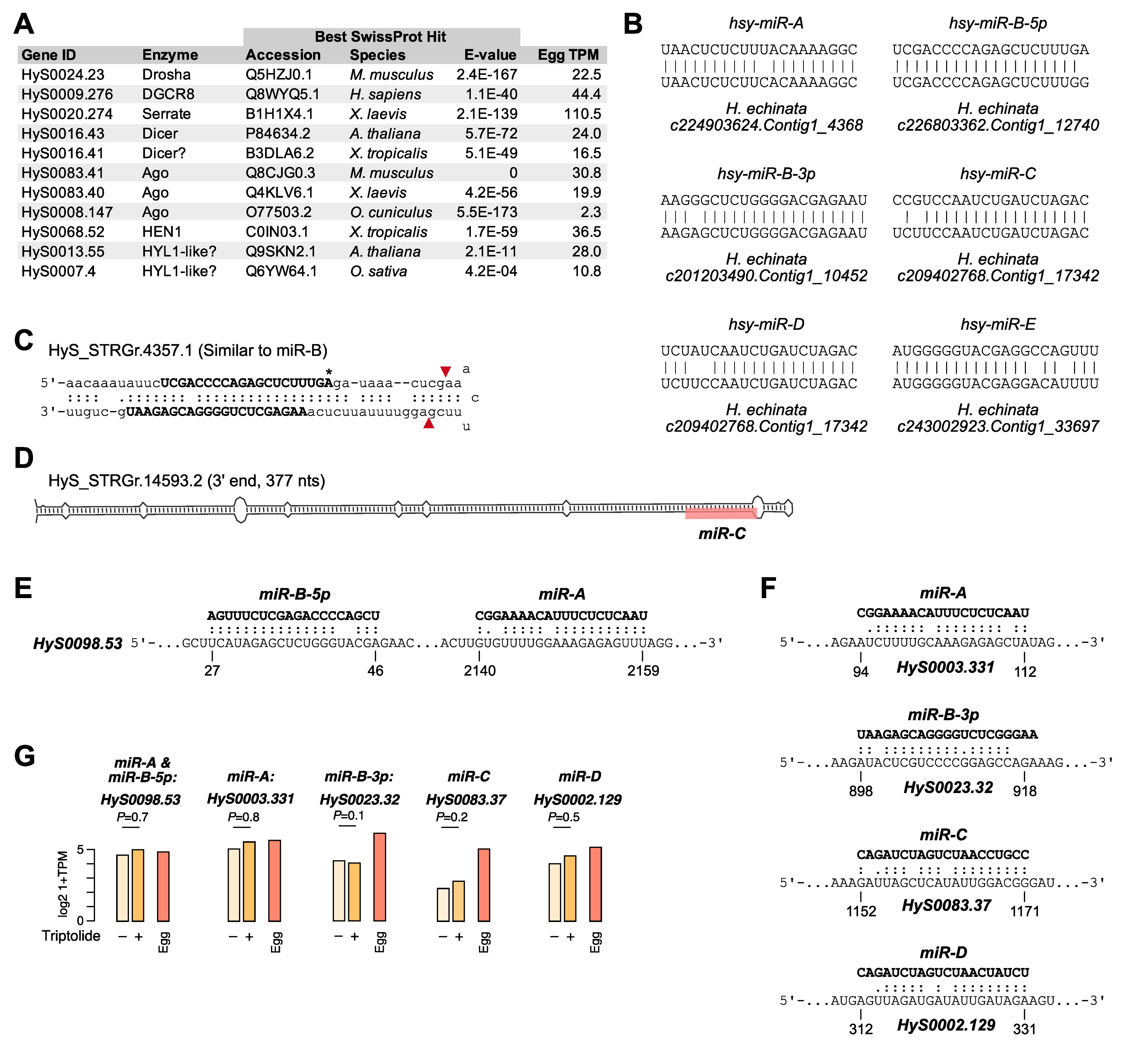

Supplement: S8 Fig — (A) Maternal expression levels for predicted components of the miRNA biogenesis pathway and the RNA-induced silencing complex. (B) Alignment of predicted H. symbiolongicarpus mature miRNAs with H. echinata miRNAs. (C) A second transcript that contains the predicted miR-B miRNA sequences, but in a structural context inconsistent with canonical Dicer processing (canonical Dicer cleavage sites marked by red arrows). Asterisk marks a base difference compared to H. echinata. (D) The full duplex structure in which predicted miRNA miR-C is found, suggesting it is not a Drosha substrate. (E, F) Predicted mRNA targets, as recovered by BLAST sequence similarity, showing potential base pairing configuration with the mature miRNAs. (G) RNA-seq expression levels of predicted mRNA targets of miRNAs in the presence or absence of Triptolide, as compared to maternal levels in the egg. None of the differences are significant, by DESeq2 with FDR adjustment. (TIF) [file pgen.1010845.s008.tif]
