# Supplementary material for: Parallels and contrasts between the cnidarian and bilaterian maternal-to-zygotic transition are revealed in Hydractinia embryos
Source: PLoS Genet. 2023 Jul 13;19(7):e1010845. doi: 10.1371/journal.pgen.1010845 (PMC10368294; doi:10.1371/journal.pgen.1010845)
Supplement: Attachment — Submitted filename: Hydractinia_MZT_final.pdf [file pgen.1010845.s020.pdf]

Manuscript ID: PGENETICS-D-23-00533
Parallels and contrasts between the cnidarian and bilaterian maternal-to-zygotic transition are revealed in Hydractinia embryos

Summary
This study investigates the transcriptional biology of early embryogenesis in Hydractinia symbiolongicarpus. By comparing two methods for RNA isolation – polyA selection and ribodepletion – and pairing these analyses with pharmacological inhibition of zygotic transcription, the authors present convincing evidence for a complex suite of mechanisms regulating stabilization and clearing of maternal RNAs before and during activation of the zygotic genome. Additionally, their data suggest Hydractinia may use a novel method for ZGA involving an early wave of histone gene expression and support a potential role for microRNAs in clearing maternally deposited transcripts during ZGA. Publication of this work will contribute a strong foundation for future studies aimed at functional investigation the evolution of MZT in cnidarians and other early-diverging animal lineages. My only real criticism is with the packaging of the story. As written, this manuscript reads like a list of things that can be learned from a sequencing experiment with some rather shallow/cursory discussion of the relationship of the results to other published studies of non-bilaterians. The work is technically sound but would benefit from some deeper synthesis.

General comments:
Generally, the conclusions/summary statements presented for each paragraph in the results section are rather poorly developed. In most cases, they simply restate the results with no additional insight or depth. This makes the paper longer than it needs to be and rather difficult to read. Perhaps one way to fix this is to propose some specific hypotheses derived from analysis of other taxa (particularly non-bilaterian taxa) and then to describe the ways in which the observations made in Hydractinia support or refute those predictions. The authors should also consider integrating the results and discussion for each section, rather than segregating the discussion at the end. As written, the discussion section lacks depth and is largely redundant with the results.

Specific comments:
Throughout – The authors should be more precise in their language. “Activation” is confusing – sometimes maternally deposited genes are referred to as “activated” (which would mean polyadenylated, I guess?), other times this term refers to genes undergoing de novo transcription from the zygotic genome. Likewise, “genome activation” is implied at 2hpf when 20 histones are de novo transcribed and also at 4hpf when the first transcripts with introns are detected. The timing of genome activation should be precisely defined. The designation of “significantly activated” as applied to transcripts seems to have different quantitative values throughout (>1TPM in some places and >5TPM in others). This needs to be clarified throughout the manuscript with justifications for why different cutoffs were chosen for each type of analysis.
Abstract - “…regulatory activities analogous to bilaterians” – this is too vague to have any meaning.

Figure 5 – define “early-cleared” and “late-cleared” in the caption
Figure 6 – some of these miRNAs appear to have expression prior to ZGA (before 4hpf – Fig 6B right panels) and are only incompletely knocked down in Triptolide treatment. Doesn’t this imply that some of them are maternally deposited? This should be addressed.
Figure 7 – this figure isn’t terribly informative as it simply restates the results. It would be more valuable to present a summary showing a comparison of Hydractinia to other taxa. One effective way to do this could be to present a cladogram of model organisms in which MZT has

been investigated annotated to indicate which taxa use each of the mechanisms described in this study. This would make it very clear to the reader which mechanisms were likely present in the common ancestor of animals (or the common ancestor of cnidarians and bilaterians) vs which traits likely evolved later (e.g., in the ancestor of vertebrates).

Line 60 – concurrent with what? ZGA? Maternal transcript readenylation?
Line 64 – "RNA-binding proteins…" don't we already know this about Nematostella?
Line 81 – "..at a phylotypic stage…" this doesn't really make sense as written. The concept of a phylotypic stage is also highly controversial and invoking it here doesn't add anything to the story. I suggest removing this comment. If you choose to keep it, please define it.
Line 84-87 – "thus, the magnitude and timing…" I would encourage the authors to use caution here in implying that what you learn from Hydractinia will tell us anything about "the cnidarian condition". Hydractinia and Nematostella may do completely different things as these animals diverged over 600MYA and embryogenesis may be highly divergent in hydroids.
Line 113 – "suggesting that ancestral gene regulatory paradigms…" what does this mean?
Line 136 – these 20 genes are all histones, which is very cool! Are these 20 also captured in the 471 upregulated genes from the polyA dataset or are they not adenylated? Do you expect this to be a unique aspect of ZGA in Hydractinia or do you think we will find similar upregulation of histones in other non-bilaterians if we use a ribo-depletion approach? More synthesis on this super cool result, please! Also, delayed genome activation isn't really the exciting result here – the cool observation is that only 20 pioneer genes (all histones) are upregulated before any other part of the zygotic genome! Wow! What are they doing? Are they repressive? Are they permissive? Good place for speculation.
Line 171 – I don't know what to make of this…20 histones are upregulated at 2h, but most genes with introns aren't transcribed until 4h? What's going on here? When do you see the first transcription factors transcribed from the zygotic genome? Several gastrulation-specific transcription factors are mentioned but I suspect that's far too late to represent the first TFs expressed from ZGA.
Line 238 – What is the logic behind reporting the combined TPM of multiple genes? Doesn't this just overinflate the inferred value of any one of these genes? This should be explained
Line 259 – "Repetitive and transposon-related…" It's not clear to me what this section contributes to our understanding of MZT. Can this be moved to the methods to streamline the story?
Line 292 – Can stage-specific gene expression arise before genome activation? This section seems a bit arbitrary, especially since the target genes (Sox and POU) are maternally loaded and don't fit the pattern suggested in the heading anyway. Can this section be reduced or removed? Or can you provide some deeper context for why it is important for these Sox and POU genes to be maternally deposited. What do they do in Hydractinia? Why would those processes need to be underway before ZGA?
Line 354 – "…Triptolide treatment…" it appears some genes were activated in this experiment – what are they? What's your interpretation? Is this just a leaky experiment or are you showing RNA polymerase-independent transcription dynamics? This experiment is cool and deserves some deeper analysis!
Line 396 – what is a "primary transcript"?
Line 420 – define "significantly upregulated"
Line 438 – "Thus, …" this is not very insightful. Do any animals lack cytoplasmic polyadenylation and maternal clearance? And didn't we already know that delayed genome activation is a shared feature of cnidarians and bilaterians (and ctenophores, for that matter)?
Line 448 – "In the future…" what is "metabolic labeling and what will this contributed to our understanding of MZT? Why do we need more sensitivity?

Line 452 – "..as well as…" this again just repeats the results – transposons might be involved. So what? How might they be involved and what would that tell us? Why is this important to mention?
Line 455 – this comparison of tandemly repeated histones and tandemly repeated miRNAs is lost on me. Why are you comparing miRNAs that presumably clear maternal products with histone RNAs which presumably facilitate activation of zygotic gene transcription? Also, why is their tandem genomic organization important? Is this the configuration with the lowest steric hinderance, thus requiring the minimum amount of energy to increase expression before onset of zygotic transcription? Please expound.
Line 493 - Typo? Sox3 is a group C sox gene and Sox2 is a group B sox gene. It's not accurate to call Sox3 a Sox2 homolog.
