# Supplementary material for: Parallels and contrasts between the cnidarian and bilaterian maternal-to-zygotic transition are revealed in Hydractinia embryos
Source: PLoS Genet. 2023 Jul 13;19(7):e1010845. doi: 10.1371/journal.pgen.1010845 (PMC10368294; doi:10.1371/journal.pgen.1010845)
Supplement: Attachment — Submitted filename: Ayers23 - Response to reviewers.docx [file pgen.1010845.s021.docx]

We thank all 3 reviewers for their supportive comments and suggestions for improvement. We have now made many edits, described below:

**Reviewer #1: The manuscript by Ayers et al is an important contribution, significantly expanding our understanding of early embryonic mechanisms to an understudied taxon. Mechanisms that control the maternal to zygotic transition in animals are varied and likely have evolved quickly. Therefore, studies in cnidarians, which occupy a critical phylogenetic position, are important for understanding the common ancient mechanisms from which extant mechanisms evolved. While a handful of studies have addressed this issue superficially in cnidarians, this is the most comprehensive study to date. The study is well conceived, carefully performed and analyzed, and the manuscript is extremely well written.

I have only a small number of comments, all of which are minor:

Figure 1: I would find it helpful to have a diagram of Hydractinia development that correlates the hours post fertilization, the cell number, and the embryonic stage. (Line 93: Is the blastula really 16 cells?)**

We have added a new Fig 1A to summarize developmental time versus cell number, which are based on the work of Kraus...Frank 2014. Regarding the 16-cell blastula, Kraus et al report the emergence of a small blastocoel cavity at 16 cells. At 32-cell stage, the cells have already segregated into two layers, endodermal inside of ectodermal, marking the start of gastrulation.

**Figure 5A: I completely understand the point being made in figures 5A-C, but I’m really struggling to understand the specific information being conveyed in the Figure 5A bubble plot. I don’t understand what each square represents in terms of comparing the two data sets. It is also unclear what the colors mean. I think this is the one figure panel in the whole paper that wasn’t immediately clear and could use some updating.**

We have revised Fig 5A and the legend to hopefully better emphasize that most cleared genes experience deadenylation, as measured by poly(A)+ RNA-seq, before degradation.

**I was very curious about the H4K20 methyltransferase findings! Are H4K20 methyltransferases expanded in other hydroids, or more broadly in the cnidarian phylum? It would be helpful to better understand how these data into the context of the animal tree. Even though the use of pharmacological inhibitors comes with a lot of caveats, it could be interesting to see how the SETD8 inhibitor used in the referenced Hydra study affects MZT in Hydractinia.**

Thanks for the great suggestion -- we indeed find expansion of putative H4K20 methyltransferases in Hydra, but not in Nematostella, so it may be a shared hydroid but not pan-cnidarian characteristic. In doing our due diligence for the model bilaterians, we actually found that sea urchin also has a large expansion of H4K20 methyltransferases, with similar early expression dynamics as Hydractinia and strong BLAST sequence similarity as well! These results are presented in new Supp Figs S3-4.

We definitely would like to follow up with the SETD8 inhibitor in the future (we wish we could include that experiment in this manuscript, but we have logistical challenges currently).

**Reviewer #2**

**Summary**

**This study investigates the transcriptional biology of early embryogenesis in Hydractinia symbiolongicarpus. By comparing two methods for RNA isolation – polyA selection and ribodepletion – and pairing these analyses with pharmacological inhibition of zygotic transcription, the authors present convincing evidence for a complex suite of mechanisms regulating stabilization and clearing of maternal RNAs before and during activation of the zygotic genome. Additionally, their data suggest Hydractinia may use a novel method for ZGA involving an early wave of histone gene expression and support a potential role for microRNAs in clearing maternally deposited transcripts during ZGA. Publication of this work will contribute a strong foundation for future studies aimed at functional investigation the evolution of MZT in cnidarians and other early-diverging animal lineages. My only real criticism is with the packaging of the story. As written, this manuscript reads like a list of things that can be learned from a sequencing experiment with some rather shallow/cursory discussion of the relationship of the results to other published studies of non-bilaterians. The work is technically sound but would benefit from some deeper synthesis.**

**General comments:**

**Generally, the conclusions/summary statements presented for each paragraph in the results section are rather poorly developed. In most cases, they simply restate the results with no additional insight or depth. This makes the paper longer than it needs to be and rather difficult to read. Perhaps one way to fix this is to propose some specific hypotheses derived from analysis of other taxa (particularly non-bilaterian taxa) and then to describe the ways in which the observations made in Hydractinia support or refute those predictions. The authors should also consider integrating the results and discussion for each section, rather than segregating the discussion at the end. As written, the discussion section lacks depth and is largely redundant with the results.**

We appreciate the suggestions. In our experience, some readers do find it helpful when results are reiterated at the end of each results section. We ended up keeping the discussion separate, but have attempted to tighten the results summaries and emphasize the insights, while also peppering the results sections with some of these insights as well.

**Specific comments:**

**Throughout – The authors should be more precise in their language. “Activation” is confusing – sometimes maternally deposited genes are referred to as “activated” (which would mean polyadenylated, I guess?), other times this term refers to genes undergoing de novo transcription from the zygotic genome. Likewise, “genome activation” is implied at 2hpf when 20 histones are de novo transcribed and also at 4hpf when the first transcripts with introns are detected. The timing of genome activation should be precisely defined.**

We use the term "activation" to mean transcriptional activation, which is consistent with the field, and we now explicitly say this (Line 156). Increases in RNA-seq levels attributed to polyadenylation are described without using the words activate/activation. Genes with a maternal contribution can subsequently be transcriptionally activated again in the embryo, which may be a source of confusion -- we have ensured that this is clear in the revised manuscript. For clarity, we have removed the one use of the term "post-transcriptional activation" from the introduction.

When referring to the timing of genome activation, one can distinguish when the first newly transcribed genes are detected, versus when transcription is detected over a large number of genes, by some definition of large. In some species such as mouse and zebrafish, there are explicitly defined and mechanistically separable "minor" and "major" waves of genome activation, but in other taxa we could just be observing a slow ramp up of activation that includes more and more genes over time. We have added a sentence to the intro to clarify this (Line 69).

**The designation of “significantly activated” as applied to transcripts seems to have different quantitative values throughout (>1TPM in some places and >5TPM in others). This needs to be clarified throughout the manuscript with justifications for why different cutoffs were chosen for each type of analysis.**

We use "significant" to always mean statistically significant according to differential expression analysis by DESeq2. We use 1 TPM as the threshold for an mRNA being present (applies mostly to the maternal contribution, and not with respect to a differential expression analysis), and 5 TPM to distinguish more highly expressed mRNA of interest (usually related to a differential expression analysis). We have added this explanation to the Methods (Line 711).

**Abstract - “...regulatory activities analogous to bilaterians” – this is too vague to have any meaning.**

We've modified the sentence to read "regulatory activities similar to many bilaterians"

**Figure 5 – define “early-cleared” and “late-cleared” in the caption**

These are defined in the caption: "Top panels show genes whose clearance was detected 2-4 h.p.f. in poly(A)+ RNA-seq (early cleared), bottom panels ≥5 h.p.f. (late cleared)."

**Figure 6 – some of these miRNAs appear to have expression prior to ZGA (before 4hpf – Fig 6B right panels) and are only incompletely knocked down in Triptolide treatment. Doesn’t this imply that some of them are maternally deposited? This should be addressed.**

It's likely the Triptolide is incompletely effective (Supp Fig S5), but it is true that there may be some maternal contribution for these putative miRNA transcripts. Also note that the log2 scale emphasizes low expression levels. We've added a statement in the results (Line 471) and the discussion (Line 597)

**Figure 7 – this figure isn’t terribly informative as it simply restates the results. It would be more valuable to present a summary showing a comparison of Hydractinia to other taxa. One effective way to do this could be to present a cladogram of model organisms in which MZT has**

**been investigated annotated to indicate which taxa use each of the mechanisms described in this study. This would make it very clear to the reader which mechanisms were likely present in the common ancestor of animals (or the common ancestor of cnidarians and bilaterians) vs which traits likely evolved later (e.g., in the ancestor of vertebrates).**

We've added a figure panel (Fig 7b) summarizing our findings in the context of other animals

**Line 60 – concurrent with what? ZGA? Maternal transcript readenylation?**

Reworded to say "Many maternal mRNA will also undergo regulated clearance..."

**Line 64 – “RNA-binding proteins...” don’t we already know this about Nematostella?**

There is evidence for activation of a Smaug homolog in Nematostella embryos (Helm 2013), which we cite on line 443, though we're not aware of additional evidence for Nematostella RBPs participating in maternal clearance

**Line 81 – “..at a phylotypic stage...” this doesn’t really make sense as written. The concept of a phylotypic stage is also highly controversial and invoking it here doesn’t add anything to the story. I suggest removing this comment. If you choose to keep it, please define it.**

We've rephrased it to say "implying that both genome activation and maternal clearance are occurring at similar early embryonic stages to many egg-laying bilaterians."

**Line 84-87 – “thus, the magnitude and timing...” I would encourage the authors to use caution here in implying that what you learn from Hydractinia will tell us anything about “the cnidarian condition”. Hydractinia and Nematostella may do completely different things as these animals diverged over 600MYA and embryogenesis may be highly divergent in hydroids.**

We've change this to say "any cnidarian"

**Line 113 – “suggesting that ancestral gene regulatory paradigms...” what does this mean?**

That was poor word choice. It was probably supposed to say "ancient" but we've changed this to read "suggesting that shared gene regulatory paradigms underlie early embryogenesis programs across the animal tree."

**Line 136 – these 20 genes are all histones, which is very cool! Are these 20 also captured in the 471 upregulated genes from the polyA dataset or are they not adenylated? Do you expect this to be a unique aspect of ZGA in Hydractinia or do you think we will find similar upregulation of histones in other non-bilaterians if we use a ribo-depletion approach? More synthesis on this super cool result, please! Also, delayed genome activation isn’t really the exciting result here – the cool observation is that only 20 pioneer genes (all histones) are upregulated before any other part of the zygotic genome! Wow! What are they doing? Are they repressive? Are they permissive? Good place for speculation.**

Thank you, we think it's pretty cool too. Replication-dependent histones are not well captured by poly(A)+ RNA-seq, though they do show up as activated eventually in our poly(A)+ samples. The genes up in the 2 h.p.f. poly(A)+ dataset do not include any of the histone genes.

Yes, ribo depletion would be needed in other taxa to accurately measure histone expression using RNA-seq. After further literature searches, we found an old result in mouse embryos (Graves et al, PNAS 1985) that observes a sizeable maternal contribution of histone mRNA in the egg by dot blot, which is rapidly degraded (along with most maternal mRNA) before the first cleavage. Levels recover by 32 cells, indicating that histone genes are activated at some point, though it's unlikely to be among the first genes -- in mouse, the first activated genes are mostly transposon related.

We speculate about their roles in the discussion (Line 540 onward). Most other taxa go from high maternal to lower embryonic histone levels, which correlates with repressive to permissive. We think they might be repressive in Hydractinia, to establish cell-type specific gene expression patterns.

**Line 171 – I don’t know what to make of this...20 histones are upregulated at 2h, but most genes with introns aren’t transcribed until 4h? What’s going on here? When do you see the first transcription factors transcribed from the zygotic genome? Several gastrulation-specific transcription factors are mentioned but I suspect that’s far too late to represent the first TFs expressed from ZGA.**

We think histones are the earliest because (mechanistically) there's something special about their regulation. Could be the genomic organization, could be their short length -- we speculate in the Discussion (Line 518). We see the first TFs starting at 4 h.p.f. (Supp Fig S6).

**Line 238 – What is the logic behind reporting the combined TPM of multiple genes? Doesn’t this just overinflate the inferred value of any one of these genes? This should be explained**

In retrospect, reporting the summed TPM may not have been very useful -- it was to put this class of genes in context of the other genes activated at 4 h.p.f. We've removed the summed TPM value for simplicity.

**Line 259 – “Repetitive and transposon-related...” It’s not clear to me what this section contributes to our understanding of MZT. Can this be moved to the methods to streamline the story?**

We've removed some of the details, which were already described in the methods. There is a connection between repetitive sequences and transposons with ZGA that we originally did not sufficiently explain, but have expanded upon now.

**Line 292 – Can stage-specific gene expression arise before genome activation? This section seems a bit arbitrary, especially since the target genes (Sox and POU) are maternally loaded and don’t fit the pattern suggested in the heading anyway. Can this section be reduced or removed? Or can you provide some deeper context for why it is important for these Sox and POU genes to be maternally deposited. What do they do in Hydractinia? Why would those processes need to be underway before ZGA?**

The figure in question shows maternally contributed TF mRNA that decrease in later stages, hence their egg stage-specific expression. We've reworded the section header and edited the section to make this clearer. We predict certain maternal TFs will be required for genome activation, similar to other taxa. Sox and POU genes play this role in zebrafish and Xenopus, and are more broadly associated with vertebrate pluripotent stem cells. And a Hydractinia POU TF plays a role in adult stem cells. We've revised this section to emphasize all of this more.

**Line 354 – “...Triptolide treatment...” it appears some genes were activated in this experiment – what are they? What’s your interpretation? Is this just a leaky experiment or are you showing RNA polymerase-independent transcription dynamics? This experiment is cool and deserves some deeper analysis!**

Many mRNA that are normally lower at 7 h.p.f. due to post-transcriptional regulation (i.e. maternal clearance) will have higher levels in the absence of transcription, if their regulator fails to be transcribed. So this would account for the bulk of the higher signal (which we of course rely on in our Fig 5 analyses). However, the two prominently higher genes in the orig Supp Fig S5 (now Supp Fig S7) HyS0422.6 and HyS4764.1 are both single-exon genes similar to Retrovirus-related reverse transcriptase genes, suggesting that these might actually be transposon sequences and not transcribed by Pol II. Indeed, Triptolide does not seem to affect Pol III transcript levels in Hydractinia (Supp Fig S5B). Conversely, these transposons may be inhibited by mechanisms requiring genome activation, which would account for their up-expression upon Triptolide treatment. This is all speculation of course, which we now include in the figure legend.

**Line 396 – what is a “primary transcript”?**

Canonical microRNAs are transcribed as long "primary miRNA" transcripts. We adjusted the wording to explicitly say the term

**Line 420 – define “significantly upregulated”**

Rephrased to be "significantly higher levels"

**Line 438 – “Thus, ...” this is not very insightful. Do any animals lack cytoplasmic polyadenylation and maternal clearance? And didn’t we already know that delayed genome activation is a shared feature of cnidarians and bilaterians (and ctenophores, for that matter)?**

After we submitted our manuscript, a preprint was posted that demonstrated cytoplasmic polyadenylation in *M. leidyi* and Nematostella embryos (Rouhana et al, bioRxiv 2023), but before that we did not know. Delayed genome activation is likely in *M. leidyi* and Nematostella, but in both studies, expression level increases were observed between the first two time points in the time courses; in the absence of consecutive RNA-seq timepoints that demonstrate no change in RNA levels, we don't definitively know if there is a quiescent period.

**Line 448 – “In the future...” what is “metabolic labeling and what will this contributed to our understanding of MZT? Why do we need more sensitivity?**

Metabolic labeling with e.g. 4SU is used for pull-down of nascent transcripts, which has previously been used to increase detection sensitivity of activated genes in the embryo against a large maternal contribution. If most of the RNA-seq read depth is going toward maternal mRNA, it reduces the likelihood of recovering lowly transcribed genes. We've clarified this in the discussion (Line 510).

**Line 452 – “..as well as...” this again just repeats the results – transposons might be involved. So what? How might they be involved and what would that tell us? Why is this important to mention?**

We've explained further in the "Repetitive and transposon-related RNA..." results section and the discussion

**Line 455 – this comparison of tandemly repeated histones and tandemly repeated miRNAs is lost on me. Why are you comparing miRNAs that presumably clear maternal products with histone RNAs which presumably facilitate activation of zygotic gene transcription? Also, why is their tandem genomic organization important? Is this the configuration with the lowest steric hinderance, thus requiring the minimum amount of energy to increase expression before onset of zygotic transcription? Please expound.**

It is indeed the genomic organization and not the functions of the transcribed genes that is interesting to us, in light of results in zebrafish that show the tandemly duplicated mir-430 locus forms a transcription body and is activated first (Hadzhiev et al, Dev Cell 2023). We have augmented the discussion to emphasize this more

**Line 493 - Typo? Sox3 is a group C sox gene and Sox2 is a group B sox gene. It’s not accurate to call Sox3 a Sox2 homolog.**

Our understanding is that vertebrate Sox1, 2, and 3 are Group B1 sox genes (Bowles et al, Dev Biol 2000, Kamachi & Kondoh, Development 2013; among others)

**Reviewer #3: This manuscript describes a study of embryonic development in the model cnidarian Hydractinia symbiolongicarpus with a focus on activation of the zygotic genome and the dynamics of the maternal mRNA pool present in the egg. This study is of considerable significance because it represents the first thorough and carefully designed investigation of these phenomena in a non-bilaterian organism. As the sister taxon to bilaterians, Cnidaria is a particularly important phylum for carrying out studies of embryonic development. Comparisons of developmental processes in Cnidaria and Bilateria allow us to define how these processes were established at a particularly important stage in the evolution of metazoans.

The authors show that the zygotic genome is activated at the 64 cell stage. They also show that Hydractinia embryos carry out the same maternal mRNA readenylation, deadenylation, and clearance as is seen in the embryos of model bilaterians such as the frog Xenopus laevis. Interestingly, the activation of the zygotic genome is dominated by activation histone gene expression.

The data in this paper are extensive, are of high quality, and are carefully analyzed. Because of the ability to generate transgenic lines, and to edit genes in Hydractinia with CRISPR/Cas, this study has the additional important feature of setting the stage for future detailed functional studies

I have only one concern about the experimental approaches used in this study. The authors make use of Triptolide, which they describe as an inhibitor of RNA Polymerase II. My quick search of the literature indicates that Triptolide has a number of targets in addition to RNA Polymerase II. Does this create difficulties in interpretation of the data generated from the experiments using Triptolide? I would like to see this issue discussed in the paper.**

Triptolide has previously been used as an alternative to alpha amanitin to inhibit genome activation and downstream developmental processes in zebrafish and Xenopus embryos (Chan et al, Dev Cell 2019, Kontur et al, Cell Rep 2020; Gibeaux et al, Nature 2018), which we now cite. The literature is hazy regarding which of the other reported targets of Triptolide may just be downstream consequences of transcription inhibition, but we now acknowledge in the Discussion the possibility that an interaction between Triptolide and a calcium channel Pkd2 (the main other reported target of Triptolide) could have an impact on mRNA stability (Line 586)
